# Functional glycomics and evidence for gain- and loss-of-functions of target proteins for glycosyltransferases involved in *N*-glycan biosynthesis: their pivotal roles in growth and development, cancer metastasis and antibody therapy against cancer

**Published:** 2004-02-01

**Authors:** Naoyuki Taniguchi, Jianguo Gu, Motoko Takahashi, Eiji Miyoshi

**Affiliations:** Department of Biochemistry, Osaka University Graduate School of Medicine, 2-2, Yamadaoka, Suita, Osaka 565-0871

**Keywords:** Remodeling of sugar chains, glycosyltransferase, gain- and loss-of-functions, cancer metastasis, growth and development, antibody therapy

## Abstract

*N*-Acetylglucosaminyltransferase V (GnT-V), *N*-acetylglucosaminyltransferase III (GnT-III) and ***α***1-6 fucosyltransferase (Fut8) catalyze reactions that form biologically important branching *N*-linked sugar chains in glycoproteins. The above three branching *N*-glycan sugar chains, ***β***1-6 GlcNAc branching, bisecting GlcNAc and core fucose (***α***1-6 fucose), play major roles in cancer invasion and metastasis, the inhibition of cancer metastasis, and antibody-dependent cellular cytotoxicity (ADCC), growth and development, respectively. A functional glycomic approach identified the gain- and loss-of-functions of glycoproteins as the result of the aberrant glycosylation. A membrane-type metal dependent serine proteinase designated matriptase which contains ***β***1-6 GlcNAc branching became resistant to auto-digestion and proteolysis by trypsin, and resulted in a constitutively active form which might be implicated in cancer invasion and metastasis. GnT-V also acts as an angiogenic factor without the mediation of functions as a glycosyltransferase. Recently, a GnT-V homologue, GnT-IX has been identified. This gene is expressed at relatively high levels in the brain and acts on *N*-glycans to form a unique branched structure, as well as *O*-mannosyl glycans. The addition of bisecting GlcNAc to various signaling molecules or adhesion molecules suppresses cancer metastasis. Fut8 knock-out mice, due to the lack of a core fucose (***α***1-6 fucose) in target glycoproteins, show disorders in growth and development. The presence of a bisecting GlcNAc or the absence of a core fucose in IgG molecules enhances ADCC activity for killing tumor cells by up to 10 to 100 fold and therefore is thought to have considerable use in antibody therapy against cancer. These data clearly indicate that gain- and loss-of-functions of target proteins for these glycosyltransferases are biologically important.

## Introduction

The post genomic era, after completion of the sequencing of the human genome and that of many other species, has opened new avenues for our understanding of the genome net-work including interactions of protein-protein and protein-DNA, structure and function of proteins, and the post-translational modification of proteins which may be directly linked to certain diseases. It is well known that a large number of proteins undergo posttranslational modifications with corresponding changes to their structure and function. Among the various posttranslational modification reactions of proteins, glycosylation is the most abundant, nearly 50% of all proteins are considered to be glycosylated. 1) Glycosylation reactions are catalyzed by the catalytic action of glycosyltransferases and sugar chains are added to various complex carbohydrates such as glycoproteins, glycolipids and proteoglycans.2) Sugar chains play an important role in cell growth, cell-cell interactions, cancer metastasis, development, differentiation, and immune reactions.3) A large number of glycosyltransferases have been cloned and some of their important functions are now understood. Fringe is a glycosyltransferase that regulates Notch signaling4),5) and the mutation of ***β***1-2 *N*-acetylglucosaminyltransferase (POMGnT1) results in Muscle Eye Brain disease. 6) Various congenital disorders of glycosylation (CDG) are due to the mutation of glycosyltransferases or related genes7),8) and various glycosyltransferase gene KO mice have been developed.9),10) To identify the actual target proteins that may be directly related to the cause of the diseases is an important issue because phenotypic changes or symptoms of CDG patients are sometimes not a direct consequence of glycosyltransferase gene deletion or mutations but an indirect consequence due to the aberrant glycosylation of target glycoproteins or complex carbohydrates. In this context, our goal is to identify target proteins that carry a specific sugar chain by using sugar remodeling techniques and functional glycomics.11)

This review summarizes the gain- and loss-of-functions of branching sugar chains produced by three glycosyltransferases that are involved in *N*-linked glycan biosynthesis.

## Purification of glycosyltransferases and the cloning of their genes

The authors’ group has been interested in tumor-associated changes in glycoproteins such as *γ*-glutamyltranspeptidase which plays a major role in the biosynthesis of glutathione12),13) and found that the bisecting GlcNAc residues are found in enzymes purified from ascites hepatoma AH-66 but not in enzymes purified from normal rat livers.14) In order to confirm the mechanism that controls the appearance of the bisecting GlcNAc residues in the enzymes purified from tumor cells and to identify the specific glycosyltransferase responsible for producing the bisecting GlcNAc, we developed assay methods for various glycosyltransferases including *N*-acetylglucosaminyltransferases III, IV and V using pyridylaminated sugar chains15) as an acceptor substrate.16),17) This assay method, which is simple and reproducible, permitted the assay of enzyme activities on a small scale and was suitable for use during the purification of unstable enzymes. Using this method, we successfully purified GnT-III, by affinity chromatography using an acceptor substrate as a ligand.18) This type of affinity chromatography was originally developed and utilized by Hill’s group19) using donor and acceptor molecules as ligands.

**Fig. 1:**
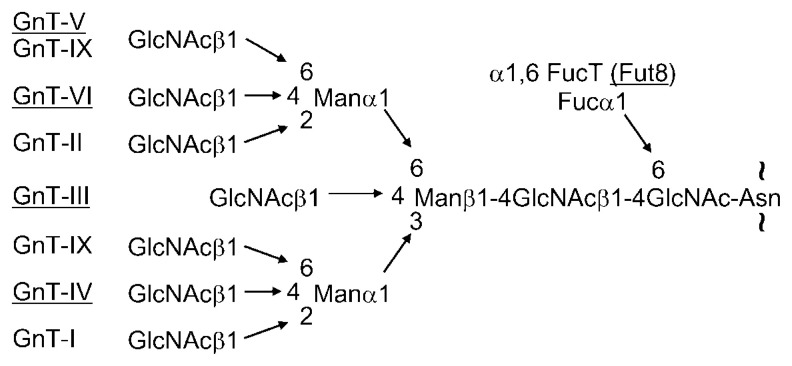
Five glycosyltransferases (*underlined*) purified by our group and for which their genes were cloned. These enzymes determine the branching structure of N-linked sugar chains that will be further biosynthesized. GnT-I and GnT-II were purified and their genes were cloned by other groups. In this review we focus mainly on the three enzymes such as GnT-III, GnT-V, and Fut8.

After obtaining partial amino acid sequences, we were able to clone the cDNA for GnT-III.18) Subsequently, this strategy was applied to the other enzymes such as GnT-V20),21) GnT-IV,22) GnT-VI,23),24) the central type I synthetic enzyme,25) and ***α***1-6 fucosyltransferase (Fut8)26),27) (Fig. 1). All these enzymes have essentially few sequence homologies with each others, therefore it was necessary to purify the enzyme proteins to obtain partial amino acid sequence data and to clone the cDNAs. Here we demonstrate some new functions of sugar chains by a sugar remodeling technique using above cloned glycosyltransferase genes.

### Gain-of-function of GnT-V

The ***β***1-6 GlcNAc branching structure acquires the properties of cancer invasion and metastasis. Five glycosyltransferase genes cloned by our group have a type II transmembrane topology and are tightly bound to the Golgi apparatus. Their genes have multiple promoters and are expressed in an organ and tissue-specific manner.2) Various glycosyltransferases provide sugar chains with structural diversity. The relation between GnT-V and cancer metastasis has been reported by Dennis *et al*.28) and Yamashita *et al*.29) as judged by lectin reactivity and structure analyses. Our group also reported on the upregulation of GnT-V in the liver of a rodent model of hepatocarcinogenesis as well as regenerative liver.30),31) However, the mechanism by which glycosyltransferase genes are regulated remains unclear. We reported on the direct implication of a transcription factor, Ets-1, in the underlying mechanism of the gene expression of GnT-V in various cancer cell lines32)–34) and up-regulation of GnT-V by TGF-***β***35) or Her2/neu was also reported.36) The level of GnT-V mRNA in human hepatoma tissues is correlated well with that of Ets-1 mRNA. A dominant negative form of Ets-1 was reported to suppress GnT-V expression.34) These data confirm that the GnT-V gene is generally regulated by Ets-1. It has recently been reported that several glycosyltransferase genes are regulated by Ets-1 and some other glycosyltransferases appear to be regulated by a similar mechanism. These results may provide a new route for elucidating the mechanism of cancer metastasis via Ets-1.

A different type of underlying mechanism for cancer metastasis may be operative but the acquisition of ***β***1-6 GlcNAc branching of a specified glycoprotein(s) may cause functional changes in metastatic potential.37)–39) We established a gastric cancer cell line which overexpressed GnT-V and identified a matriptase, a membrane type metal dependent serine proteinase, as a target glycoprotein of GnT-V.40),41) The matriptase was independently cloned by two groups and shown to be involved in cancer progression, invasion and metastasis. 42),43) Matriptase is also involved in the activation of hepatocyte growth factor (HGF) and tissue plasminogen activator.44) It has 4 potential *N*-glycosylation sites, and one of which, Asn 772 is the actual target amino acid residue for glycosylation by GnT-V as evidenced by site-directed mutagenesis.41) Asn 772 is conserved only in family members of serine proteinases such as trypsin etc. Matriptase in which ***β***1-6 GlcNAc branching is added by GnT-V was found to be resistant to auto-digestion by itself as well as exogenously added trypsin. Thus, the aberrant glycosylation of matriptase by GnT-V at *N*-glycosylation sites such as Asn 772 led to a constitutively active form of this proteinase and this may enhance susceptibility to cancer invasion and metastasis.

We have also found that GnT-V is a bifunctional protein and functions as a glycosyltransferase as well as an angiogenic releasing factor.45) GnT-V has a heparin binding domain, KRKRKK which is very similar to that of other angiogenic factors. Deletion mutants of GnT-V which lack catalytic activity showed angiogenic activity which can be attributed to its competitive binding to certain proteoglycans with FGF2, indicating that the angiogenic factor releasing properties of GnT-V is not involved in its catalytic properties. Moreover we found that GnT-V is cleaved by proteinase(s) including *γ*-secretase and then released as a soluble form, which acts as an angiogenic releasing factor.

Very recently our group identified a new enzyme designated GnT-IX, a ***β***1-6 *N*-acetylglucosaminyltransferase, a homolog of GnT-V, as shown in Fig. 1.46) Subsequently Pierce’s group also reported on the same enzyme.47) Our group showed that GnT-IX also acts on *O*-mannosylglycans. 48) This report is very important because the enzyme is specifically expressed in the brain and testis. Moreover, there is an enzyme designated POMGnT-1 which catalyses the addition of ***β***1-2 GlcNAc to *O*-mannosylglycan and the mutation of which causes MEB (muscle eye brain) disease due to an impairment in the glycosylation of **α** dystroglycans.6),49) Even though GnT-IX is not expressed in muscle tissues, the lack of GnT-IX may be implicated in the neuropathy and the development of KO mice of GnT-IX may answer the question of whether this enzyme is involved in some types of brain or neural diseases.

## Gain-of-function of GnT-III: bisecting GlcNAc has anti-metastatic function

We hypothesized that the suppression of ***β***1-6 GlcNAc branching by the inhibition of GnT-V activity would be possible, based on substrate specificity studies. Since GnT-III and GnT-V act on the same substrate but once GnT-III acts on the substrate first and produces a bisecting GlcNAc structure,20) GnT-V is not able to act further. We therefore assumed that this may lead to a suppression of metastatic potential *in vivo*. Computer modeling of these reactions provided support for the above hypothesis. 50) Based on these analyses, we transfected the GnT-III gene into melanoma B16F1 cells with a high metastatic potential, and attempted to remodel the sugar chains. When the GnT-III gene-transfected melanoma cells were injected into syngeneic mice via the tail vein, lung metastasis was minimal51) whereas many lung metastatic foci were observed in mock-transfected melanoma cells. Sugar analyses by lectin blotting of the cells indicated that the ***β***1-6 GlcNAc branching structures originally found in the parental cells were no longer present in the GnT-III transfectants. Moreover, some of the glycoproteins in the GnT-III transfectants would be expected to be functionally modified as judged by cell biological parameters such as cell adhesion and cell invasion capacity. Among those molecules, E-cadherin a homophilic type of adhesion molecule52) which is highly associated with the prevention of metastasis53) was aberrantly glycosylated in the GnT-III gene transfectants. The E-cadherin on cell surfaces was found to be resistant to proteolysis and remained on the cell-cell borders as result of sugar remodeling. As a result of these changes, cancer cells with metastatic potential were not able to be detached from the cells54) to proceed to the metastatic machinery. We also found that the glycosylation of E-cadherin leads to a reduced level of phosphorylation of ***β***-catenin by EGFR (EGF-receptor) or src signaling and therefore ***β***-catenin maintains a tight complex with E-cadherin and is not translocated into the nuclei.55) ***β***-Catenin otherwise enhances various gene expressions that are related to cell growth or oncogenesis. Suppression of the phosphorylation of ***β***-catenin, therefore, permits it to remain on the cell surface and not to be released from the complex and this may also enhance the homophilic interactions of E-cadherin and contribute to the suppression of cancer metastasis.

Transfection of the GnT-III gene also results in the alteration of functions of growth factor receptors. The authors’ group, in collaboration with the Moskal group, found that GnT-III modulates the functions of epidermal growth factor receptors.56) Moreover, GuT-III modification of EGFR enhances mitogen-activated protein (MAP) kinase activity.57) This may lead to biological changes in processes such as cell growth and apoptosis. On the other hand, regarding growth factors, it is known that nerve growth factor facilitates the differentiation of PC12 cells into neurite cells. An overexpression of the GnT-III gene in PC12 cells resulted in the addition of a bisecting GlcNAc to Trk A, a nerve growth factor (NGF) receptor, and even when NGF was added to the cells, the dimerization of the receptor was inhibited and the signaling for differentiation was suppressed. 58) Moreover, an Asn420Gln mutant of the EGF receptor resulted in the spontaneous oligomerization and activation of the receptor, both in the presence and absence of the EGF ligand.59)

More recently Shibukawa *et al.*60) also reported that the transfection of GnT-III in Hela cells suppresses H_2_O_2_-induced activation of the PKC***δ***-JNK pathway, resulting in the inhibition of apoptosis. These lines of evidence indicate that bisecting GlcNAc is capable of gaining function in terms of intracellular signaling.

## Loss-of-function of Fut8: growth retardation due to the lack of a core fucose

**Fig. 2:**
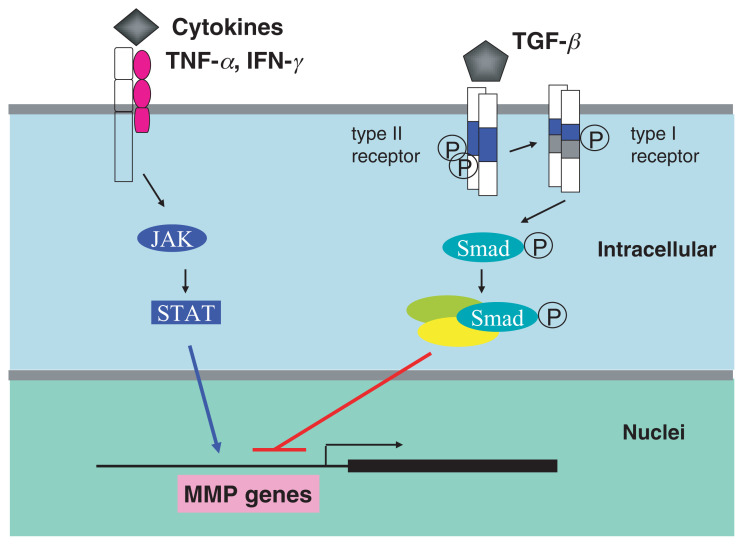
Positive and negative regulation of MMP genes.

Fut8 catalyzes the transfer of a fucose residue from GDP-fucose to position 6 of the innermost GlcNAc residue of the hybrid and complex types of *N*-linked oligosaccharides on glycoproteins. 61) The gene that encodes this enzyme was designated Fut8. The enzyme was first purified to homogeneity and cloned by our group from porcine tissue 26) as well as from human gastric cancer cells.27) The deletion of the Fut8, brought about marked phenotypic changes of mice. Namely, 60% of the mice died during the neonatal period and the remainder died within 3 weeks. The mice suffered from lung emphysema. An examination of respiratory capacity indicated that Fut8 KO mice that had been exposed to hypercapnea or hypoxia had an extremely low ventilation capacity, compared to heterozygous or normal control mice. It has been reported that there are several causes which may lead to lung emphysema. The overexpression of platelet-derived growth factor (PDGF), matrix metalloproteinase (MMPs) −1, −2, −9 and −12 has been reported to be causes of human lung emphysema.62)–66) Interestingly, in Fut8 KO mice, the expression levels of some MMPs were aberrantly up-regulated, while tissue inhibitors of metalloproteinases (TIMPs) were down-regulated, suggesting that imbalanced-metabolism of extracellular matrix occurred in the lung of KO mice. MMP genes are usually positively or negatively regulated by cytokines such as IFN-*γ*,67) TNF-***α***68) and IL-1365) or TGF-***β*** signaling,66)–69) respectively. Namely, IL-13 and IFN-*γ* up-regulate MMP gene expression via JAK and STAT signaling whereas TGF-***β*** down-regulates the gene expression of MMPs via the TGF-***β*** receptor and smad proteins (Fig. 2). Therefore, it would be interesting to determine whether the ***α***1-6 fucosylation of membrane receptors regulates their biological functions.

## Gain-of-function of bisecting GlcNAc and core fucose in the ADCC activity

**Fig. 3:**
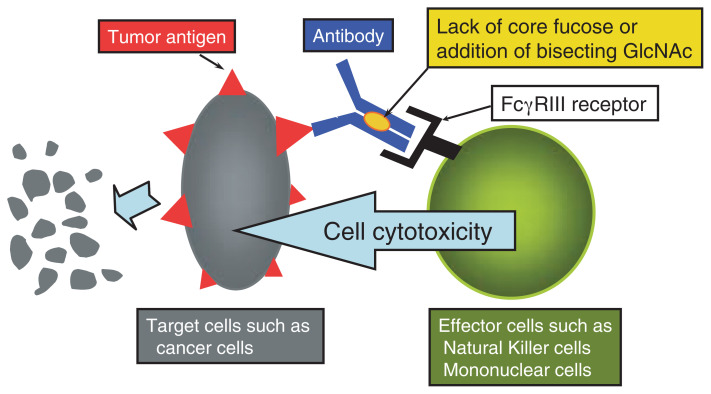
Antibody dependent cellular cytotoxicity (ADCC) of IgG1 and the role of bisecting GlcNAc and core fucose (The figure was taken from the courtesy from Dr. M. Sato, Kyowa Hakko Kogyo Co. Ltd.). The addition of bisecting GlcNAc or deletion of core fucose in the Asn 297 in the Fc portion of IgG1 enhances ADCC activity by up to 10–100 fold.

Natural Killer (NK) cells are defined by their ability to kill cells that display a foreign antigen such as tumor cells regardless of MHC type and regardless of previous sensitization (exposure) to the antigen. Our group previously reported the GnT-III transfectant of K562 cells became resistant to NK cells, suggesting that bisecting GlcNAc may play a role in NK cell-mediated cytotoxicity.70) In contrast to NK cells, cytotoxic T cells contain receptors for the Fc domain of IgG, bind to the Fc portion of an IgG antibody on the surface of target cells such as tumor cells and release cytolytic components that kill the target cell. This mechanism of killing is referred to as antibody-dependent cellular cytotoxicity (ADCC) (Fig. 3).71) A number of mechanisms for the anti-tumor activities of therapeutic antibodies have been proposed and include extended half-life, the blockage of signaling pathways, activation of apoptosis and effector-cell-mediated cytotoxicity. Clynes *et al.*72) reported that Fc*γ* receptors on effector cells are major components of the *in vivo* activity of antibodies against tumors. ADCC activity is, therefore, considered to be the major activity in antibody therapy against tumors and it is well known that mouse monoclonal antibodies and humanized antibodies, clinically effective agents such as trastuzumab (Herceptin ®) and rituximab (Rituzan ®), require both activation via Fc*γ*RIII and inhibition via Fc*γ*RIIB antibody receptors. Mice that are deficient in activating Fc receptors as well as in the binding of Fc to these receptors were unable to arrest tumor growth *in vivo*.72) These results demonstrate that Fc-receptor-dependent mechanisms are substantial contributors to the action of cytotoxic antibodies against tumors and indicate that an optimal antibody against tumors would preferentially bind to activated Fc receptors and minimally to the inhibitory partner Fc*γ*RIIB. Umana *et al.*73) reported that the expression of antibodies with altered glycoforms, especially the addition of bisecting GlcNAc leads to an increase in ADCC through a higher affinity for Fc*γ*RIII of up to 10–20 fold. They concluded that the increase in ADCC activity is therefore probably due to an increased affinity of the modified antibody for the Fc*γ*RIII receptor. A similar study was also independently reported by a Swiss group.74)

Along a similar line of evidence as described by the above two groups, Shields *et al*.75) using Lec13 cells, a variant Chinese hamster ovary cell line, produced human IgG1 that was deficient in the core fucose attached to the Asn 297-linked carbohydrate but was otherwise similar to that produced in normal Chinese hamster ovary cell lines and that from human serum. The lack of fucose on the IgG1 had no effect on its binding to human Fc*γ*RI, C1q, or the neonatal Fc receptor. The binding of the fucose-deficient IgG1 to human Fc*γ*RIIIA was enhanced by up to 50-fold.75) ADCC assays using purified peripheral blood monocytes or NK cells from several donors showed an enhanced cytotoxicity, which was particularly evident at lower antibody concentrations. Independently, Shinkawa *et al.*76) reported that the deletion of the core fucose from the IgG1 molecule enhances ADCC activity by up to 50–100 fold. This indicates that the core fucose is an important sugar chain in terms of ADCC activity. Therefore, Fut8 KO mice, which lack the core fucose in glycoproteins, may be useful for the production of various human antibodies against tumor cells. These findings clearly indicate that the ***α***1,6-fucosylation of *N*-glycans modifies the function of the glycoproteins. The strategy reported by the above reports is applicable to optimizing the ADCC activity of other therapeutic IgGs.

## Future perspectives

***β***1-6 branching GlcNAc produced by GnT-V plays a major role in cancer invasion and metastasis through stabilization of proteases and the angiogenic releasing factor. Bisecting GlcNAc and the core fucose, products of GnT-III and Fut8, respectively, have unique functions in terms of the suppression of cancer metastasis and ADCC activity against cancer and both types of branching sugar chains play a major role in cancer therapy. Gain- and loss-of-functions of various glycosyltransferase could be clarified in all glycosyltransferases identified and so far the real *in vivo* target proteins can be identified.
